# Towards National Surgical Surveillance in the UK – A Pilot Study

**DOI:** 10.1371/journal.pone.0047969

**Published:** 2012-12-11

**Authors:** Riaz Agha

**Affiliations:** 1 National Institute of Health and Clinical Excellence Scholar, London, United Kingdom; 2 Cambridge University Hospitals NHS Foundation Trust, Cambridge, United Kingdom; California Pacific Medicial Center Research Institute, United States of America

## Abstract

**Objective:**

The Bristol heart inquiry in the United Kingdom (UK) highlighted the lack of standards for evaluating surgical performance and quality. In 2009, the World Health Organisation (WHO) proposed six standardised metrics for surgical surveillance. This is the first study to collect and analyse such metrics from a cohort of National Health Service (NHS) Trusts in England, helping to determine their feasibility and utility in measuring surgical performance, its impact on public health and mortality, and for tracking surgical trends over time.

**Methods:**

Freedom of Information Act 2000 (FOI) requests for WHO standardised surgical metrics were made to 36 NHS Trusts in England during July to November 2010. Additional data on Hospital Standardised Mortality Ratio (HSMR), Patient Safety Score and Abdominal Aortic Aneurysm (AAA) volume and mortality was obtained from Dr Foster Health and *The Guardian* Newspaper. Analysis was performed using mixed-effect logistic regression.

**Results:**

30/36 trusts responded (83%). During 2005–9, 5.4 million operations were performed with a 24.2% increase in annual number of operations. This rising volume within hospitals was associated with lower mortality ratios. A 10% increase in operative volume was associated with a lower day of surgery death rate (DDR OR = 0.94, p = 0.056) and post-operative inpatient 30-day mortality (PDR30 OR = 0.93, p = 0.001). For every 10,000 more operations that an NHS Trust does, a 4% drop in PDR30 mortality was achieved. A 10% increase in the volume of elective AAAs was associated with lower elective AAA (OR = 0.96, p = 0.032) and emergency AAA (OR = 0.95, p = 0.009) PDR30 mortality. Lower DDR mortality was noted for emergency AAA mortality (OR = 0.95, p = 0.025) but not elective AAAs (OR = 0.97, p = 0.116).

**Conclusion:**

Standarised surgical metrics can provide policy makers and commissioners with valuable summary data on surgical performance allowing for statistical process control of a complex intervention. This study has shown their collection is feasible albeit using FOI and the first to show a statistically significant volume-outcome relationship for surgery as a whole within hospitals. It adds weight to the argument that patients are safer in larger hospitals or those that become larger by growing their patient base. Together with other measures, such metrics can help build a picture of surgical surveillance in the UK and potentially lead us to safer surgery.

## Introduction

Public health surveillance has long relied on standardised metrics to quantify disease burden in a population, track mortality rates and guide health system programming, assessment and investment. For over half a century, such standard metrics have included; maternal mortality, infant mortality and life expectancy. Vaccination rates and treatment coverage for specific infectious diseases (e.g. Human Immunodeficiency Virus infection) are also being added to this growing pool. Cardiac surgeons have led the way in the open and transparent publication of named surgeon mortality results [1]. However, there are currently no standardised metrics for surgical surveillance globally [2]. Such metrics would allow for the assessment of the safety of perioperative care and surgical performance.

Globally an estimated 234 million major surgical operations are performed annually [3]. This volume of procedures is thought to result in seven million complications and one million deaths - double the number of annual maternal deaths and resulting in 164 m disability adjusted life years (DALYs) [1]. The average American will now undergo 9.2 surgical procedures in a lifetime and modern surgery includes everything from coronary artery bypass grafting to joint replacement and transplantation [4].

The public inquiry into children's heart surgery at the Bristol Royal Infirmary in the UK [5] drew attention to the lack of standards for evaluating and tracking surgical performance in the NHS and for assessing the quality of care. The need to monitor standards and benchmark outcomes in healthcare has been underscored more recently by several inquiries in the UK including; the Shipman inquiry [6], the Mid-Staffordshire inquiry [7] and reports into Basildon and Thurrock hospitals by the UK hospital regulator - the Care Quality Commission (CQC) [8].

In the UK, over the last 10 years surgical waiting lists have come down and the focus has shifted towards quality and safety of care [9]. This focus is being shared more globally and in 2007, the World Health Organisation (WHO) launched an initiative: *“Safe Surgery Saves lives”*. One aspect of the programme was to develop standardised measures for surveillance of the volume of surgical care and its effect on public health outcomes over time. A technical working group consisting of experts in epidemiology, global health and surgical outcomes from around the world came together to develop standardised metrics for assessing surgical services [2]. The group proposed six as follows (see Table 1.)

**Table 1:** The six standardised surgical metrics together with their definitions and rationales (adapted from Weiser et al's original paper^2^).

Metric	Definition	Rationale
Annual number of operations (process measure)	The absolute number of all surgical procedures, defined as the incision, excision, or manipulation of tissue that requires regional or general anaesthesia, or profound sedation to control pain, undertaken in an operating room	Surgical volume is an indication of the access to and use of health care, particularly surgical services
Number of operating rooms (structure measure)	Operating rooms are rooms used specifically for surgical procedures and equipped to deliver anaesthesia	The number of operating rooms available to a population is a structural indicator of the ability to provide surgical interventions
Number of accredited surgeons (structure measure)	Accredited surgeons are physicians who have achieved certification in a surgical specialty as recognised by the accepted national standards of the member state or national professional organisations. Consultant surgeons were used for the purposes of this study.	The availability and composition of human resources for health is an important indicator of the strength of the health system
Number of accredited anaesthesia professionals (structure measure)	Accredited anaesthesia professionals are physicians, nurses, and other practitioners who have achieved certification in the provision of anaesthesia as recognised by the accepted national standards of the member state or national professional organisations. Consultant Anaesthetists were used for the purposes of this study.	The availability and composition of human resources for health is an important indicator of the strength of the health system
Day-of-surgery death ratio (DDR) (outcome measure)	Number of deaths on the day of surgery, irrespective of cause, divided by the number of surgical procedures in a given year or period, reported as a percentage	Day-of-surgery death ratios allow the health system to assess its performance and the state of health of the population
Postoperative in-hospital death ratio limited to 30 days (PDR30) (outcomes measure)	Number of deaths in the hospital following surgery, irrespective of cause and limited to 30 days, divided by the number of surgical procedures done in a given year or period, reported as a percentage	Understanding the in-hospital death ratio after surgery provides insight into the risks associated with surgical intervention

These standardised surgical metrics provide structure, process and outcome measures for evaluating healthcare [10]. The WHO states that such data when combined with other routinely collected data could provide a baseline that can then be used to monitor surgical services on an annual basis. Thus allowing policy makers to quantify demand for surgical services, surgical safety, identify access barriers, track mortality rates, benchmark outcomes, provide an early warning system for poor performance and potentially assess the effects of new interventions. It would also provide Government, the NHS and healthcare authorities with a rich dataset that can be used to programme and reshape health services nationally.

The objective of this study was to retrospectively gather and analyse these standardised surgical metrics from a cohort of NHS Hospitals in England. This study would help assess the practical feasibility of gathering such metrics and help determine their utility in measuring surgical performance and its impact on public health and mortality, and for tracking surgical trends over time.

## Methods

A sample of 36 NHS Trusts in England, representing 23% of Acute and Foundation NHS Trusts, were approached in late 2010 using a Freedom of Information (FOI) Act 2000 [11] request for yearly data on the six standardised surgical metrics for the period 2005–9. NHS Trusts are in effect Public Sector Corporations consisting of one or more hospitals responsible for delivering care on behalf of the English NHS [12]. NHS Trusts are designed to serve the need of the local population and have variable budgets and catchment areas. The Freedom of Information (FOI) Act was passed on 30 November 2000 in the UK and was part of the Government's commitment to greater openness in the public sector. It gives a general right of access to all types of recorded information held by public authorities with full access granted in January 2005. Information released under FOI undergoes a number of internal checks for accuracy prior to release.

Half were randomly selected (via a mouse scroll wheel technique on the NHS Choices website: http://www.nhs.uk/Pages/HomePage.aspx) and the other half were based on a convenience sample which had been previously approached (unsuccessfully with an 11% response rate) for data by a polite letter. Following this poor response it was felt that FOI would be a more robust approach for data. For the purposes of uniformity and in keeping with local practice, ‘Accredited Surgeons and Anaesthetists’ in the WHO definition was changed to Consultant Surgeons and Anaesthetists respectively. Consultant Surgeons and Anaesthetists in the UK are those that have passed fellowship or ‘exit’ exams of the established professional body; the Royal College of Surgeons and Royal College of Anaesthetists respectively, have successfully completed their training/residency with the award of a certificate of completion of training and been appointed to a substantive role as a Consultant to a specific NHS Trust for independent delivery of care to patients (the equivalent of an Attending in the USA).

Upon receipt of the dataset from the NHS trust, the information was sent back to the Medical Director for the same trust for them to check the data and verify its accuracy. Whilst Hospital Episode Statistics (HES) could have been used to provide mortality and operative volume data, they would not have been able to provide all the data (e.g. staffing and operating rooms) and there are ongoing concerns about HES data accuracy [13], [14]. Hence a single request was thought to be more reliable and efficient and would make verification by the Medical Director simpler and more consistent.

Additional data were added from the UK broadsheet newspaper *The Guardian and its* ‘*Safety in numbers for hospital patients*’ FOI investigation on Abdominal Aortic Aneurysm (AAA) mortality [15]. This provided useful raw data that the author could analyse and incorporate into this study rather than duplicating prior efforts. Hospital Standardised Mortality Ratio (HSMR) data together with Patient Safety Score (PSS) for 2008/9 for the same NHS trusts was sourced from Dr Foster Health (a provider of healthcare information) [16]. The PSS relates to the statistical combination through z-scoring of 13 equally weighted indicators from the patient safety domain of Dr Foster's quality accounts, giving an overall measure of each hospital [17]. The result is a score between 0 and 100, with 100 being the best. Dr Foster is a joint venture between the NHS Information Centre for health and social care and Dr Foster Holdings LLP, it has a code of conduct that prohibits political bias and is independently monitored. All data was stored and basic analysis performed in Excel**®** 2007 database (Microsoft, Redmond, WA, USA) under strict password controlled access.

### Statistical Methods

All statistical models were developed in STATA v11.2 (available at www.stata.com, published by StataCorp LP, Texas, USA) and used mixed-effect logistic regression [18] incorporating both fixed and random effects [19] with p<0.05 considered significant. Models were developed to longitudinally analyse changes in the standardised surgical metrics across different hospitals and within hospitals over the five year period 2005–9. HSMR and PSS models were cross-sectional and used only 2008 data. In each case DDR was the outcome variable and the effect of increasing either HSMR or PSS by 10% was modelled. Both models contain a random effect for hospital. The capacity model (looking at volume of operations, number of surgeons and anaesthetists) uses five years of data with DDR again as the outcome. The independent effects of 10% changes in the following were modelled; volume of operations, surgeons per 10,000 operations, anaesthetists per 10,000 operations and rooms per 10,000 operations.

The models were also adjusted for year as a categorical variable. The hospital specific model includes a random intercept for hospital and a random slope for year (continuous). This model can be considered as modelling the within hospital change. The population average model does not contain any random effects but uses a sandwich estimator for standard errors and associated p-values and confidence intervals to allow for the clustering of repeated observations within hospitals. This can be considered as the between hospital effect.

The elective AAA and emergency AAA models use three years of data. For both models death rate is the outcome. The effect of a 10% increase in DDR adjusting for volume of elective AAA and year (categorical) was modelled. As above for the hospital specific models, this also includes a random intercept for hospital and a random slope for year (continuous) and the population average models use a sandwich estimator. In order to model a 10% change, the variable in question was log transformed and then divided by log (1.1) (natural logs in both cases). This transformed variable was used in the regression and the resulting odds ratio corresponded to a 10% change.

## Results

30 out of 36 NHS trusts approached responded to the FOI request with 27 providing a complete data set and three providing partial data (83% response rate). One NHS trust refused to provide data and the other five were unable to provide data despite multiple requests and reminders. Data validation letters were sent to 27 Medical Directors with a response received from 14. Of these 14, nine (64%) confirmed the data as accurate, four (29%) made adjustments to the mortality data and one (7%) refused to confirm the data.

### WHO Standardised Surgical Metrics

#### Process Measures

**Figure 1:**
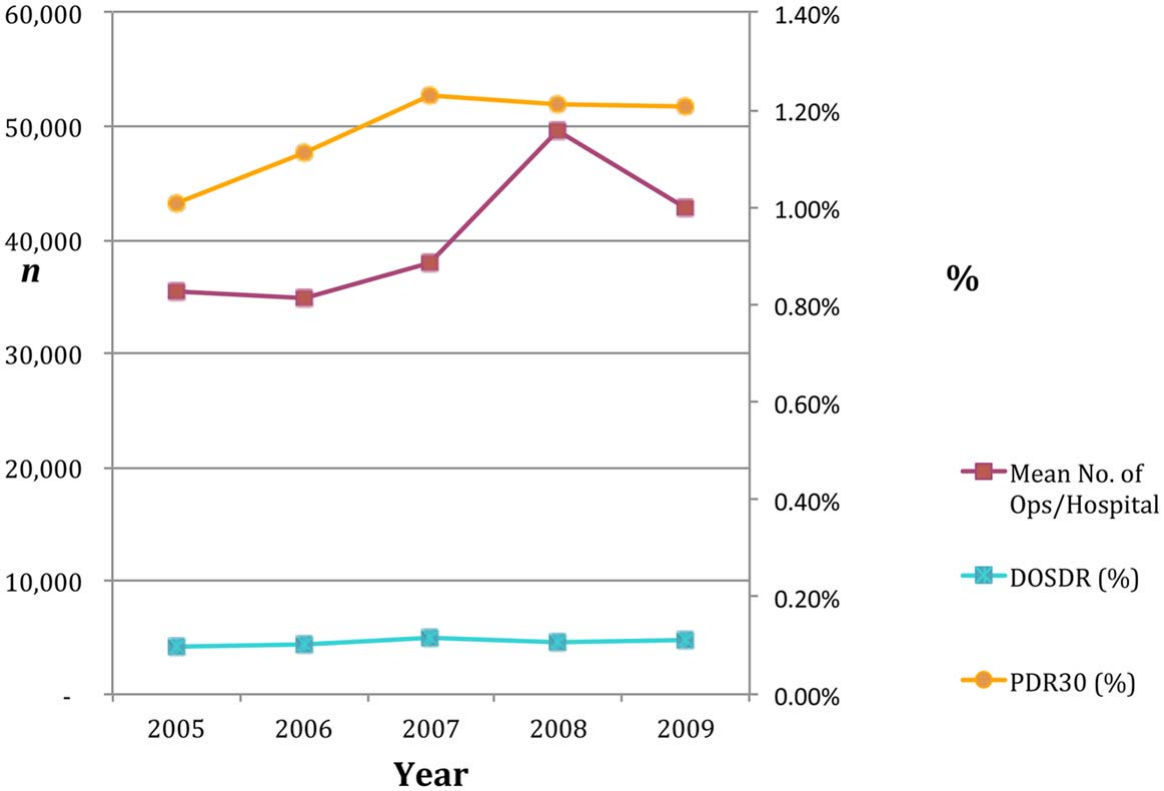
Summary of activity and mortality standardised surgical metrics.

Over the five-year period, 5.4 million operations were conducted and there was a 24.2% increase in the volume of operations over this period (figure 1 and table 2). This ranged for individual hospitals from −19.8% to +105%.

**Table 2:** Summary results for standardised surgical metrics.

Standardised Surgical Metrics	Total in 2009	Net change 2005–9
Number of Operations (27 NHS Trusts)	1,156,443 (5,396,262 total 2005–9)	24.2% increase
Number of Operating Rooms (30 NHS Trusts)	736	9.9% increase
Number of Consultant Surgeons (30 NHS Trusts)	2,497	19.3% increase
Number of Consultant Anaesthetists (30 NHS Trusts)	1,472	18.2% increase
Day of Surgery Death Ratio (27 NHS Trusts)	0.0011% mean	27.9% increase
30 day in-hospital death ratio (27 NHS Trusts)	0.012% mean	11.3% increase

#### Structure Measures

**Figure 2:**
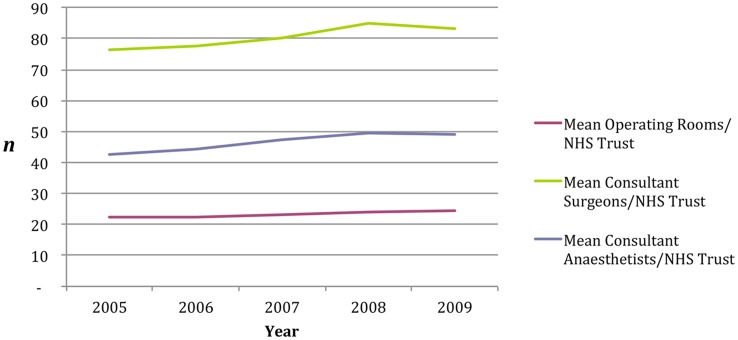
Summary of capacity and staffing level standardised surgical metrics.

The data did not show evidence to support a consistent relationship between staffing levels; Consultant Surgeons (DDR OR = 1.00, CI = 0.94–1.06, p = 0.993), Consultant Anaesthetists (DDR OR = 1.00, CI = 0.94–1.07, p = 0.929) or number of operating rooms (DDR OR = 1.00, CI = 0.95–1.07, p = 0.897) and DDR and PDR30 outcomes (figure 2).

#### Outcomes Measures

**Figure 3:**
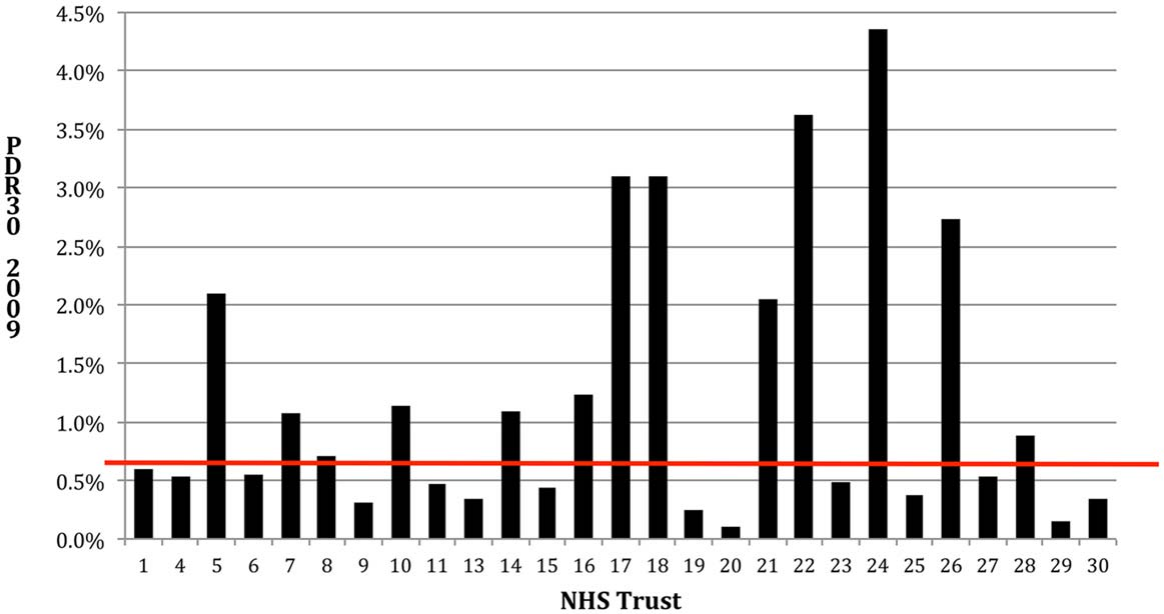
PDR30 in 2009 for NHS trusts with available data in the sample.

**Figure 4:**
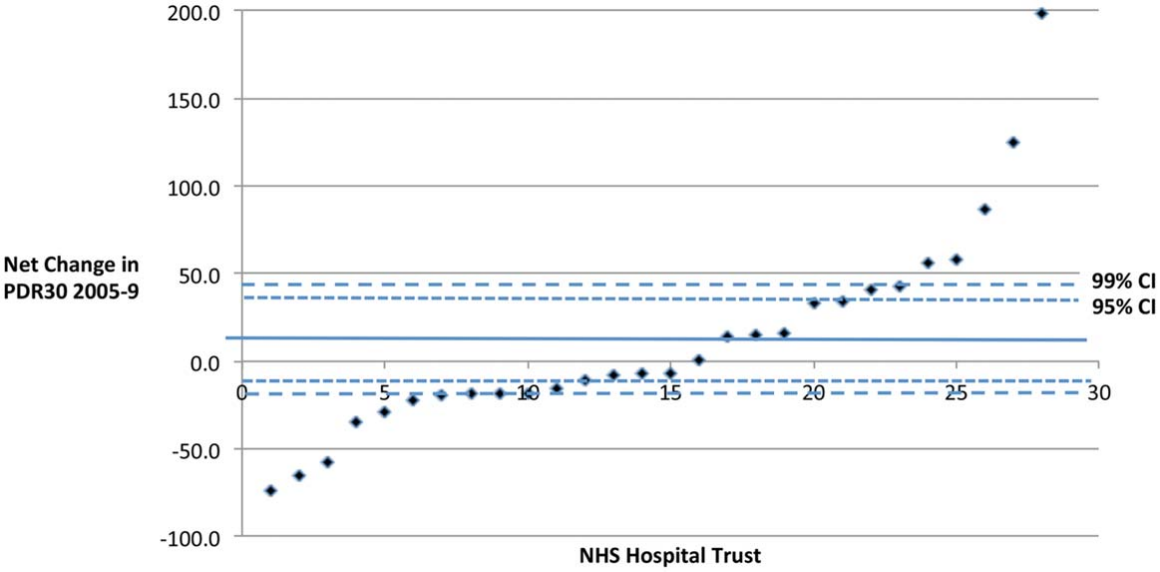
2005–9 Net change in PDR30 for each of the 26 NHS trusts for which there was complete or near complete data (NHS Trusts with two or more years of data missing were excluded).

Cross-sectionally for any particular year, there was a range of DDRs and PDR30s across the hospitals sampled (see figures 3 and 4). These ratios consistently increase in some hospitals and decrease in others over the five-year period under study. Figure 4 illustrates how outliers can be identified outside of the 95% and 99% confidence intervals.

A 10% increase in volume of operations within and across hospitals over the five-year period was associated with lower DDR (OR = 0.94, CI = 0.87–1.00, p = 0.056) and PDR30 outcomes (OR = 0.93, CI = 0.90–0.97, p = 0.001). For every 10,000 more operations that an NHS Trust does a 4% drop in PDR30 mortality was achieved.

### HSMR and PSS

There was no evidence to support a link between 2008 HSMR and surgical mortality DDR (OR = 0.95, CI = 0.73–1.23, p = 0.684) and PDR30 (OR = 0.93, CI = 0.72–1.20, p = 0.573) outcomes (modelled using a 10 point increase in HSMR). However, there was a significant association between 2008 Patient Safety Score (PSS) and surgical mortality DDR (OR = 1.18, CI = 1.03–1.35, p = 0.017) and PDR30 (OR = 1.19, CI = 1.05–1.34, p = 0.005) outcomes (modelled on a 10 point increase in PSS).

### AAA Data Analysis

A 10% increase in the volume of elective AAAs (2006–8) was associated with lower elective AAA (OR = 0.96, CI = 0.92–1.00, p = 0.032) and emergency AAA (OR = 0.95, CI = 0.91–0.99, p = 0.009) PDR30 mortality. Lower DDR mortality was noted for emergency AAA mortality (OR = 0.95, CI = 0.92–0.99, p = 0.025) but not elective AAAs (OR = 0.97, CI = 0.93–1.01, p = 0.116).

## Discussion

To the author's knowledge at the time of writing, this is the first study to collect the WHO standardised surgical metrics in the UK. Gathering such data is feasible and potentially useful but was found to be difficult without resorting to use of FOI. The demand upon surgical services increased by 24.2% over the period 2005–9 with each NHS Trust carrying out an average of 7,400 more operations in 2009 than in 2005. This trend for increasing demand is consistent with the work of Wieser et al who have shown how demand is increasing globally [1] and this is supported by the work of others [20]. Over this period staffing levels have risen in concert with the greater volume of operations. The relatively small increase in operating theatres in the face of this rise in volume is probably the result of better theatre utilisation and possible waiting list initiatives, such as elective theatre lists at weekends [21].

The finding that a 10% increase in the volume of elective AAAs was associated with lower elective and emergency AAA mortality is consistent with previous volume-outcome data already reported for specific surgical procedures like elective or urgent open AAA repair [22], [23] elective endovascular AAA repair [24] and carotid endarterectomy [25], [26]. Such data has led increasingly to the call for a national reconfiguration of vascular services into a centralised ‘hub and spoke’ model of care [27], [28] with all major arterial interventions to be performed in high volume specialist centres where all vascular surgeons present exceed minimum case volumes criteria [29]. Holt et al have also found that high volume centres are more likely to operate on ruptured AAAs [30], perhaps due to the greater number and confidence of the operating surgeons and support staff, access to the latest technology and intensive therapy unit facilities [31]. Awopetu et al found that higher-volume centres were associated with reduced amputation and mortality rates post lower limb vascular surgery although they did point to significant heterogeneity within their data [32].

The result that a 10% increase in operative volume (across all surgical specialties) within and across hospitals was associated with lower DDR and PDR30, shows that volume-outcome relationships may extend across the surgical service as a whole, not just for vascular surgery. To the author's knowledge this is the first study to show such an effect. Such data again point to the need for high volume centres and the reconfiguration of surgical services on a national basis, particularly for those operations that involve significant morbidity and mortality. However, mortality and patient safety are not the only considerations in this debate, with patient preferences, travel times and low volume centres which perform well also providing policy makers with much food for thought [33]. It is no surprise that such reconfigurations have been put on hold by the government [34].

The data also shows a significant degree of variance amongst the cohort both cross-sectionally (figure 3) and longitudinally (figure 4), particularly for PDR30.

Such data can help to determine outliers and may prompt further study of potential underlying reasons [35] especially when there is a consistent longitudinal trend. This is facilitated by the strong signal to noise ratio that high risk surgery provides [36].

Indeed, Poloniecki et al [37] examined false alarm rates and mortality at a regional cardiothoracic centre and advised gathering such data on an ongoing basis. They called for the introduction of hospital mortality monitoring groups to routinely monitor and chart all deaths in a hospital by specialty.

Aylin et al [38] did similar work in primary care where they studied 1,009 family physicians. Of these, 33 (including Harold Shipman) crossed the alarm threshold designed to detect a two standard deviation increase in standardised mortality, with a 97% successful detection and a 5% false-alarm rate. It may be plausible to look at such a system at the hospital or NHS Trust level in a prospective manner using statistical process control methodology. It could also complement and be a useful addition to the broad corpora of data that is already collected on a routine basis by agencies like the Care Quality Commission, National Patient Safety Agency, the Department of Health, the NHS, the National Audit office and the office for National Statistics.

There was no evidence of a link between HSMR and surgical mortality. In the UK, Canada, the Netherlands and the United States, HSMR has been used for many years within organisations to monitor performance and response to various quality and safety programs [39]. It has also been used by a variety of stakeholders to compare performance between hospitals [40]. The data demonstrates that HSMR should not be used as a surrogate of surgical performance or quality per se between hospitals. This may be a reflection of how HSMR is calculated and weighted. The variation in non-surgical death and surgical case-mix appears to be too large to see any direct relationship between HSMR and surgical mortality. However, increasing PSS was paradoxically linked with an increase in surgical mortality. This may reflect how this scoring is weighted more towards medical patients who outnumber surgical patients and who are more strongly represented in overall mortality figures. PSS has been abandoned by Dr Foster as a metric in its most recent hospital guides [41].

There are several limitations to this study. The sample was not fully randomised and had initially been designed to give a spread of NHS trusts across England. The mortality data was not case-mix adjusted and there is likely to be significant variation here across England. However this is much less likely to vary significantly for the same institution over the five year period for which data was collected, hence the impact on longitudinal analyses will be limited. The author agrees with the statement by Weiser et al [2] when proposing the metrics, that these are *“not metrics of quality but rather of the effect of surgery on public health and mortality, and for tracking surgical trends over time.”*

It should be noted that some NHS trusts did not have such data readily available requiring additional time to locate, summate and verify it and 10% of NHS Trusts who responded could not provide a complete set of data, missing key operational information like number of operations performed in a year and mortality rates. This may reflect the fact that such surveillance data is not routinely collected at present. This culture needs to change. The recent report; *The Higher Risk General Surgical Patient* [42] by the Royal College of Surgeons of England and the UK Department of Health advocated a national audit of outcome as one of its key recommendations and that this could be used to address high variations in outcome across the country. Since 2001, when surveillance and mandatory reporting began for Methicillin-resistant Staphylococcus Aureus (MRSA) and *Clostridium difficle* infections across the NHS, their rates have continued to fall [43]. Recent data shows how outcomes in cardiac surgery have improved since outcome data has been published [44].

WHO Standardised surgical metrics together with other measures can help build a picture of surgical surveillance in the UK, which could be repeated on an annual basis and help build an increasingly rich corpora of surveillance. The policy implications of such types of research are important. Potentially, such an exercise could provide the NHS with rich annualised data on a national level to assess surgical volume, safety, identify barriers to access, provide a baseline to benchmark and track changes over time. Such surveillance has been shown to be of value in the recent metal on metal prosthesis scandal and could have been useful in detecting the high rupture rates of Poly Implant Prothese (PIP) breast implants earlier [45], [46]. Furthermore, the establishment of such a baseline will provide a means by which the efficacy and cost-effectiveness of policy interventions can be evaluated (e.g. the effect of rationalising services to higher volume centres of excellence). This data can be mined and utilised in health services research aimed at improving surgical services and will help to guide the NHS and national health care organisations in resource allocation and the programming of services.

## Conclusion

Standarised surgical metrics could provide policy makers and commissioners with valuable summary data on surgical performance allowing for statistical process control of a complex intervention. This is the first study to shown how their collection is feasible albeit using FOI and the first to show a statistically significant volume-outcome relationship for surgery as a whole within hospitals. It adds weight to the argument that patients are safer in larger hospitals or those that grow their patient base significantly as shown by previous work in Vascular Surgery.

Such metrics when expanded and potentially combined with other data sources could provide a data armamentarium and foundation for building a ‘performance mosaic’ for surgical services in England to help monitor demand, benchmark standards, guide rationalisation of services and identify gaps in resource allocation or safety and quality. Future work in this area includes analysis of WHO metrics data in other countries, further investigation of the link between overall surgical volume and mortality outcomes with deeper statistical process control analysis at the NHS trust level. This may have an impact on the high volume rationalisation of healthcare services taking place in the UK and in other countries for trauma and major surgery.
